# 3,3′-Diethyl-1,1′-[anthracene-9,10-diylbis(oxyethyl­ene)]diimidazolium diiodide

**DOI:** 10.1107/S1600536809038379

**Published:** 2009-09-30

**Authors:** Yanhui Hou

**Affiliations:** a School of Materials Science and Engineering, Tianjin Polytechnic University, No. 63 Chenglin Road, Tianjin 300160, People’s Republic of China

## Abstract

In the title centrosymmetric compound, C_28_H_32_N_4_O_2_^2+^ 2I^−^, the two midazole rings are approximately perpendicular to the central anthracene ring system [dihedral angle = 86.6 (2)°]. The ionic units are linked into a two-dimensional network parallel to (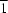
01) by C—H⋯I hydrogen bonds and π–π inter­actions involving the anthracene ring system and imidazole rings [centroid–centroid distance = 3.717 (3) Å].

## Related literature

For general background to *N*-heterocyclic carbenes and their transition metal complexes, see: Bourissou *et al.* (2000 ▶); Herrmann & Kocher (1997 ▶); Cavell & McGuinness (2004 ▶); Baker *et al.* (2004 ▶); Melaiye *et al.* (2004 ▶).
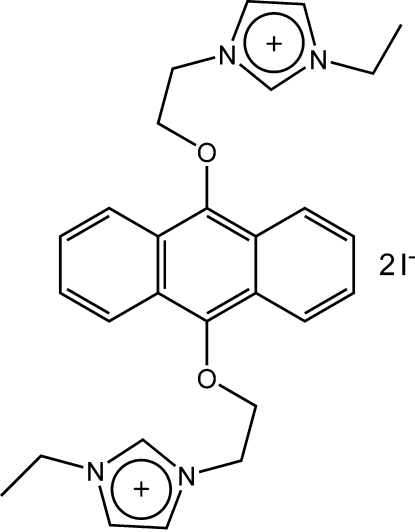


## Experimental

### 

#### Crystal data

C_28_H_32_N_4_O_2_^2+^·2I^−^
*M* *_r_* = 710.38
Monoclinic, 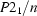

*a* = 11.4733 (13) Å
*b* = 10.6692 (12) Å
*c* = 13.1553 (15) Å
β = 112.725 (2)°
*V* = 1485.3 (3) Å^3^
*Z* = 2
Mo *K*α radiation
μ = 2.15 mm^−1^
*T* = 298 K
0.24 × 0.22 × 0.22 mm

#### Data collection

Bruker SMART CCD area-detector diffractometer
Absorption correction: multi-scan (*SADABS*; Sheldrick, 1996 ▶) *T*_min_ = 0.627, *T*_max_ = 0.650
8914 measured reflections
2603 independent reflections
1989 reflections with *I* > 2σ(*I*)
*R*_int_ = 0.035

#### Refinement

*R*[*F* ^2^ > 2σ(*F* ^2^)] = 0.044
*wR*(*F* ^2^) = 0.102
*S* = 1.07
2603 reflections
163 parameters
H-atom parameters constrained
Δρ_max_ = 0.81 e Å^−3^
Δρ_min_ = −0.27 e Å^−3^

### 

Data collection: *SMART* (Bruker, 1998 ▶); cell refinement: *SAINT* (Bruker, 1999 ▶); data reduction: *SAINT*; program(s) used to solve structure: *SHELXS97* (Sheldrick, 2008 ▶); program(s) used to refine structure: *SHELXL97* (Sheldrick, 2008 ▶); molecular graphics: *SHELXTL* (Sheldrick, 2008 ▶); software used to prepare material for publication: *SHELXTL*.

## Supplementary Material

Crystal structure: contains datablocks I, global. DOI: 10.1107/S1600536809038379/ci2907sup1.cif

Structure factors: contains datablocks I. DOI: 10.1107/S1600536809038379/ci2907Isup2.hkl

Additional supplementary materials:  crystallographic information; 3D view; checkCIF report

## Figures and Tables

**Table 1:** Hydrogen-bond geometry (Å, °)

*D*—H⋯*A*	*D*—H	H⋯*A*	*D*⋯*A*	*D*—H⋯*A*
C3—H3*A*⋯I1^i^	0.93	2.89	3.771 (5)	159
C4—H4*A*⋯I1^ii^	0.93	2.97	3.893 (5)	172
C5—H5*A*⋯I1^iii^	0.93	3.05	3.957 (6)	167

Symmetry codes: (i) 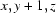; (ii) 
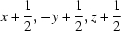
; (iii) 
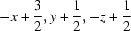
.

**Fig. 1.**
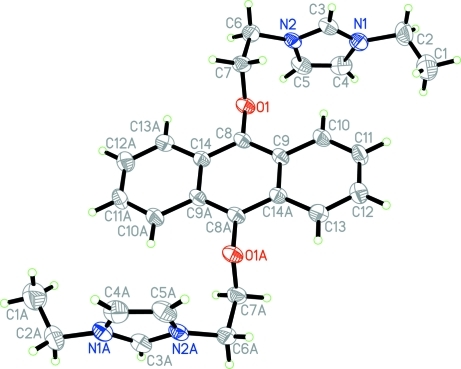
The cation of (I), showing the atom-numbering scheme. Displacement ellipsoids are drawn at the 30% probability level. Atoms labeled with the suffix A are generated by the symmetry operation (1-x, 1-y, -z).

**Fig. 2.**
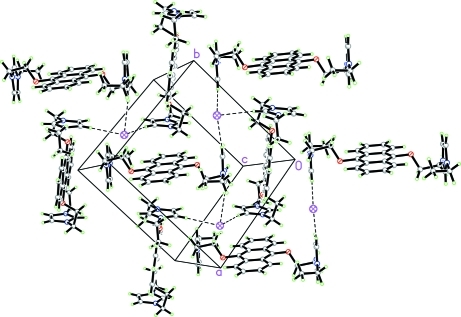
The packing diagram of (I). Dashed lines indicate C—H···I interactions.

## supplementary crystallographic information

### Comment

As ancillary ligands, N-heterocyclic carbenes (NHCs) have received considerable attention due to their strong σ-donor ability, and are attractive alternatives to the widely utilized phosphine ligands in metal coordination chemistry (Bourissou *et al.*, 2000; Herrmann & Kocher, 1997). A number of N-heterocyclic carbene transition metal complexes have been synthesized and isolated, and some of them have been successfully applied as homogeneous catalysts (Cavell & McGuinness, 2004; Baker *et al.*, 2004; Melaiye *et al.*, 2004). Herein, the synthesis and crystal structure of a new biscarbene analogue, (I), is reported.

The asymmetric unit contains one-half of the cation and a iodide anion. The cation of (I) lies across a crystallographic inversion centre (Fig. 1). The two imidazole ring planes are approximately perpendicular to the central anthracene ring system, the dihedral angle between them being 86.6 (2)°.

In the crystal, the ionic units are linked via C—H···I interactions. In addition, the anthracene ring system and imidazole rings of two adjacent molecules are stacked, with a centroid-to-centroid separation of 3.717 (3) Å indicating weak π-π interactions. The C—H···I and π-π interactions link ionic units into a two-dimensional network parallel to the (101) [Fig.2].

### Experimental

A mixture of 1,2-bis(2-chloroethoxy)anthracene (6.7 g, 20 mmol) and 1-ethylimidazole (4.22 g, 44 mmol) was refluxed in THF (100 ml) for 24 h, giving a pale yellow precipitate, which was filtered and washed with THF and recrystallized from methanol and ethyl ether. The obtained solid was dissolved in methanol (200 ml) and an aqueous solution of NH_4_I (4.64 g, 32 mmol) was added to the solution. The precipitate formed was collected by filtration and recrystallized from CH_3_CN and diethyl ether (1:6 *v*/*v*) to give the title compound (yield 95%). Analysis found: C 32.49, H 3.22, N 5.36%; calculated for C_28_H_32_N_4_O_2_I_2_: C 32.64, H 3.13, N, 5.44%. ^1^H NMR (300 *M*, *d*_6_-DMSO): *δ*9.51 (s, 2 H), 8.03 (s, 2 H), 7.97 (s, 2 H), 7.89–7.86 (m, 4 H), 7.55–7.51 (m, 4 H), 4.85 (t, *J* = 4.5 Hz, 4 H), 4.49 (t, *J* = 4.3 Hz, 4 H), 4.35 (q, *J* = 7.4 Hz, 4 H), 1.51 (t, *J* = 7.3 Hz, 6 H) p.p.m..

### Refinement

H atoms were placed in calculated positions [C-H = 0.93 (aromatic) or 0.97 Å (methylene)] and included in the final cycles of refinement using a riding-model approximation, with *U*_iso_(H) = 1.2U_eq_(carrier atom).

### Crystal data

**Table:**

C_28_H_32_N_4_O_2_^2+^·2I^−^	*F*(000) = 700
*M**_r_* = 710.38	*D*_x_ = 1.588 Mg m^−^^3^
Monoclinic, *P*2_1_/*n*	Mo *K*α radiation, λ = 0.71073 Å
Hall symbol: -P 2yn	Cell parameters from 5650 reflections
*a* = 11.4733 (13) Å	θ = 0.9–28.4°
*b* = 10.6692 (12) Å	µ = 2.15 mm^−^^1^
*c* = 13.1553 (15) Å	*T* = 298 K
β = 112.725 (2)°	Block, colourless
*V* = 1485.3 (3) Å^3^	0.24 × 0.22 × 0.22 mm
*Z* = 2	

### Data collection

**Table:**

Bruker SMART CCD area-detector diffractometer	2603 independent reflections
Radiation source: fine-focus sealed tube	1989 reflections with *I* > 2σ(*I*)
graphite	*R*_int_ = 0.035
φ and ω scans	θ_max_ = 25.0°, θ_min_ = 2.0°
Absorption correction: multi-scan (*SADABS*; Sheldrick, 1996)	*h* = −7→13
*T*_min_ = 0.627, *T*_max_ = 0.650	*k* = −12→11
8914 measured reflections	*l* = −15→15

### Refinement

**Table:**

Refinement on *F*^2^	Primary atom site location: structure-invariant direct methods
Least-squares matrix: full	Secondary atom site location: difference Fourier map
*R*[*F*^2^ > 2σ(*F*^2^)] = 0.044	Hydrogen site location: inferred from neighbouring sites
*wR*(*F*^2^) = 0.102	H-atom parameters constrained
*S* = 1.07	*w* = 1/[σ^2^(*F*_o_^2^) + (0.0505*P*)^2^] where *P* = (*F*_o_^2^ + 2*F*_c_^2^)/3
2603 reflections	(Δ/σ)_max_ = 0.001
163 parameters	Δρ_max_ = 0.81 e Å^−^^3^
0 restraints	Δρ_min_ = −0.27 e Å^−^^3^

### Special details

**Table:**

Geometry. All e.s.d.'s (except the e.s.d. in the dihedral angle between two l.s. planes) are estimated using the full covariance matrix. The cell e.s.d.'s are taken into account individually in the estimation of e.s.d.'s in distances, angles and torsion angles; correlations between e.s.d.'s in cell parameters are only used when they are defined by crystal symmetry. An approximate (isotropic) treatment of cell e.s.d.'s is used for estimating e.s.d.'s involving l.s. planes.
Refinement. Refinement of *F*^2^ against ALL reflections. The weighted *R*-factor *wR* and goodness of fit *S* are based on *F*^2^, conventional *R*-factors *R* are based on *F*, with *F* set to zero for negative *F*^2^. The threshold expression of *F*^2^ > σ(*F*^2^) is used only for calculating *R*-factors(gt) *etc*. and is not relevant to the choice of reflections for refinement. *R*-factors based on *F*^2^ are statistically about twice as large as those based on *F*, and *R*- factors based on ALL data will be even larger.

### Fractional atomic coordinates and isotropic or equivalent isotropic displacement parameters (Å^2^)

**Table:**

	*x*	*y*	*z*	*U*_iso_*/*U*_eq_	
I1	0.42215 (3)	0.07079 (3)	0.19395 (3)	0.06624 (18)	
O1	0.6771 (3)	0.6579 (3)	0.1484 (2)	0.0520 (8)	
N1	0.6819 (4)	0.7779 (4)	0.4677 (3)	0.0628 (11)	
N2	0.7451 (3)	0.8096 (3)	0.3355 (3)	0.0503 (9)	
C1	0.5148 (7)	0.6942 (7)	0.5210 (6)	0.115 (2)	
H1A	0.4719	0.7075	0.5699	0.172*	
H1B	0.5544	0.6132	0.5349	0.172*	
H1C	0.4550	0.6981	0.4461	0.172*	
C2	0.6093 (6)	0.7894 (7)	0.5390 (5)	0.100 (2)	
H2A	0.6677	0.7860	0.6155	0.120*	
H2B	0.5684	0.8708	0.5266	0.120*	
C3	0.6648 (5)	0.8459 (5)	0.3797 (4)	0.0603 (13)	
H3A	0.6055	0.9096	0.3527	0.072*	
C4	0.7780 (5)	0.6961 (5)	0.4802 (4)	0.0723 (16)	
H4A	0.8107	0.6375	0.5365	0.087*	
C5	0.8164 (5)	0.7143 (5)	0.3988 (4)	0.0651 (14)	
H5A	0.8800	0.6705	0.3868	0.078*	
C6	0.7488 (5)	0.8552 (5)	0.2318 (4)	0.0637 (13)	
H6A	0.8315	0.8392	0.2307	0.076*	
H6B	0.7342	0.9449	0.2258	0.076*	
C7	0.6479 (4)	0.7891 (4)	0.1346 (3)	0.0577 (12)	
H7A	0.5646	0.8048	0.1345	0.069*	
H7B	0.6497	0.8189	0.0656	0.069*	
C8	0.5860 (4)	0.5813 (4)	0.0729 (3)	0.0443 (10)	
C9	0.4887 (4)	0.5346 (4)	0.0997 (3)	0.0432 (10)	
C10	0.4733 (5)	0.5667 (4)	0.1992 (4)	0.0534 (12)	
H10A	0.5310	0.6204	0.2495	0.064*	
C11	0.3758 (5)	0.5200 (5)	0.2213 (4)	0.0642 (13)	
H11A	0.3676	0.5417	0.2867	0.077*	
C12	0.2873 (5)	0.4392 (5)	0.1468 (4)	0.0655 (14)	
H12A	0.2205	0.4083	0.1628	0.079*	
C13	0.2987 (5)	0.4058 (4)	0.0511 (4)	0.0557 (12)	
H13A	0.2396	0.3517	0.0027	0.067*	
C14	0.6018 (4)	0.5485 (4)	−0.0243 (3)	0.0426 (10)	

### Atomic displacement parameters (Å^2^)

**Table:**

	*U*^11^	*U*^22^	*U*^33^	*U*^12^	*U*^13^	*U*^23^
I1	0.0748 (3)	0.0667 (3)	0.0669 (2)	0.00388 (17)	0.0379 (2)	0.00174 (16)
O1	0.0521 (18)	0.0522 (19)	0.0433 (16)	−0.0023 (14)	0.0090 (14)	−0.0128 (14)
N1	0.073 (3)	0.064 (3)	0.048 (2)	−0.011 (2)	0.020 (2)	−0.013 (2)
N2	0.051 (2)	0.052 (2)	0.0430 (19)	−0.0073 (18)	0.0127 (18)	−0.0170 (17)
C1	0.124 (6)	0.129 (7)	0.115 (5)	−0.023 (5)	0.072 (5)	−0.023 (5)
C2	0.128 (6)	0.118 (5)	0.076 (4)	−0.036 (4)	0.064 (4)	−0.031 (4)
C3	0.066 (3)	0.056 (3)	0.056 (3)	0.003 (2)	0.020 (3)	−0.012 (2)
C4	0.081 (4)	0.067 (4)	0.047 (3)	0.002 (3)	0.001 (3)	0.005 (3)
C5	0.059 (3)	0.067 (3)	0.055 (3)	0.009 (3)	0.008 (3)	−0.013 (3)
C6	0.081 (4)	0.060 (3)	0.050 (3)	−0.019 (3)	0.025 (2)	−0.011 (2)
C7	0.072 (3)	0.050 (3)	0.045 (2)	−0.005 (2)	0.016 (2)	−0.007 (2)
C8	0.044 (3)	0.045 (2)	0.039 (2)	0.005 (2)	0.0102 (19)	−0.0063 (19)
C9	0.049 (3)	0.043 (2)	0.034 (2)	0.012 (2)	0.0113 (19)	−0.0012 (17)
C10	0.061 (3)	0.055 (3)	0.043 (2)	0.002 (2)	0.018 (2)	−0.007 (2)
C11	0.085 (4)	0.069 (3)	0.050 (3)	0.004 (3)	0.039 (3)	−0.007 (2)
C12	0.067 (3)	0.071 (4)	0.069 (3)	0.003 (3)	0.037 (3)	−0.005 (3)
C13	0.057 (3)	0.052 (3)	0.057 (3)	−0.001 (2)	0.021 (2)	−0.007 (2)
C14	0.043 (2)	0.040 (2)	0.041 (2)	0.0060 (19)	0.0121 (19)	−0.0009 (18)

### Geometric parameters (Å, °)

**Table:**

O1—C8	1.395 (5)	C6—H6A	0.97
O1—C7	1.434 (5)	C6—H6B	0.97
N1—C3	1.315 (6)	C7—H7A	0.97
N1—C4	1.365 (6)	C7—H7B	0.97
N1—C2	1.482 (7)	C8—C9	1.387 (6)
N2—C3	1.323 (6)	C8—C14	1.402 (6)
N2—C5	1.368 (6)	C9—C10	1.428 (6)
N2—C6	1.464 (6)	C9—C14^i^	1.432 (5)
C1—C2	1.437 (8)	C10—C11	1.355 (7)
C1—H1A	0.96	C10—H10A	0.93
C1—H1B	0.96	C11—C12	1.402 (7)
C1—H1C	0.96	C11—H11A	0.93
C2—H2A	0.97	C12—C13	1.363 (7)
C2—H2B	0.97	C12—H12A	0.93
C3—H3A	0.93	C13—C14^i^	1.407 (7)
C4—C5	1.321 (7)	C13—H13A	0.93
C4—H4A	0.93	C14—C13^i^	1.407 (7)
C5—H5A	0.93	C14—C9^i^	1.432 (5)
C6—C7	1.525 (6)		
			
C8—O1—C7	114.0 (3)	N2—C6—H6B	109.7
C3—N1—C4	107.4 (5)	C7—C6—H6B	109.7
C3—N1—C2	125.7 (5)	H6A—C6—H6B	108.2
C4—N1—C2	127.0 (5)	O1—C7—C6	106.3 (4)
C3—N2—C5	107.7 (4)	O1—C7—H7A	110.5
C3—N2—C6	126.0 (4)	C6—C7—H7A	110.5
C5—N2—C6	126.0 (4)	O1—C7—H7B	110.5
C2—C1—H1A	109.5	C6—C7—H7B	110.5
C2—C1—H1B	109.5	H7A—C7—H7B	108.7
H1A—C1—H1B	109.5	C9—C8—O1	119.0 (4)
C2—C1—H1C	109.5	C9—C8—C14	122.8 (4)
H1A—C1—H1C	109.5	O1—C8—C14	118.0 (4)
H1B—C1—H1C	109.5	C8—C9—C10	122.9 (4)
C1—C2—N1	114.2 (5)	C8—C9—C14^i^	119.0 (4)
C1—C2—H2A	108.7	C10—C9—C14^i^	118.1 (4)
N1—C2—H2A	108.7	C11—C10—C9	120.8 (4)
C1—C2—H2B	108.7	C11—C10—H10A	119.6
N1—C2—H2B	108.7	C9—C10—H10A	119.6
H2A—C2—H2B	107.6	C10—C11—C12	120.8 (5)
N1—C3—N2	109.5 (4)	C10—C11—H11A	119.6
N1—C3—H3A	125.3	C12—C11—H11A	119.6
N2—C3—H3A	125.3	C13—C12—C11	120.3 (5)
C5—C4—N1	108.3 (5)	C13—C12—H12A	119.9
C5—C4—H4A	125.8	C11—C12—H12A	119.9
N1—C4—H4A	125.8	C12—C13—C14^i^	121.2 (4)
C4—C5—N2	107.1 (5)	C12—C13—H13A	119.4
C4—C5—H5A	126.4	C14^i^—C13—H13A	119.4
N2—C5—H5A	126.4	C8—C14—C13^i^	123.0 (4)
N2—C6—C7	110.0 (4)	C8—C14—C9^i^	118.2 (4)
N2—C6—H6A	109.7	C13^i^—C14—C9^i^	118.8 (4)
C7—C6—H6A	109.7		
			
C3—N1—C2—C1	102.4 (7)	C7—O1—C8—C9	−91.0 (5)
C4—N1—C2—C1	−77.6 (7)	C7—O1—C8—C14	93.1 (4)
C4—N1—C3—N2	0.6 (5)	O1—C8—C9—C10	3.5 (6)
C2—N1—C3—N2	−179.3 (4)	C14—C8—C9—C10	179.2 (4)
C5—N2—C3—N1	−0.1 (5)	O1—C8—C9—C14^i^	−177.3 (3)
C6—N2—C3—N1	174.7 (4)	C14—C8—C9—C14^i^	−1.6 (7)
C3—N1—C4—C5	−0.9 (6)	C8—C9—C10—C11	179.1 (4)
C2—N1—C4—C5	179.0 (5)	C14^i^—C9—C10—C11	−0.1 (6)
N1—C4—C5—N2	0.8 (6)	C9—C10—C11—C12	−0.2 (8)
C3—N2—C5—C4	−0.5 (5)	C10—C11—C12—C13	0.5 (8)
C6—N2—C5—C4	−175.2 (4)	C11—C12—C13—C14^i^	−0.4 (7)
C3—N2—C6—C7	−80.2 (6)	C9—C8—C14—C13^i^	−179.1 (4)
C5—N2—C6—C7	93.6 (5)	O1—C8—C14—C13^i^	−3.3 (6)
C8—O1—C7—C6	173.3 (4)	C9—C8—C14—C9^i^	1.6 (6)
N2—C6—C7—O1	−60.2 (5)	O1—C8—C14—C9^i^	177.3 (3)

Symmetry codes: (i) −*x*+1, −*y*+1, −*z*.

### Hydrogen-bond geometry (Å, °)

**Table:**

*D*—H···*A*	*D*—H	H···*A*	*D*···*A*	*D*—H···*A*
C3—H3A···I1^ii^	0.93	2.89	3.771 (5)	159
C4—H4A···I1^iii^	0.93	2.97	3.893 (5)	172
C5—H5A···I1^iv^	0.93	3.05	3.957 (6)	167

Symmetry codes: (ii) *x*, *y*+1, *z*; (iii) *x*+1/2, −*y*+1/2, *z*+1/2; (iv) −*x*+3/2, *y*+1/2, −*z*+1/2.

## References

[bb1] Baker, M. V., Brown, D. H., Haque, R. A., Skelton, B. W. & White, A. H. (2004). *J. Chem. Soc. Dalton Trans.* pp. 3756–3764. 10.1039/B408741K 15510303

[bb2] Bourissou, D., Guerret, O., Gabbai, F. P. & Bertrand, G. (2000). *Chem. Rev.* **100**, 39–91. 10.1021/cr940472u 11749234

[bb3] Bruker (1998). *SMART* Bruker AXS Inc., Madison, Wisconsin, USA.

[bb4] Bruker (1999). *SAINT* Bruker AXS Inc., Madison, Wisconsin, USA.

[bb5] Cavell, K. J. & McGuinness, D. S. (2004). *Coord. Chem. Rev.* pp. 248–671.

[bb6] Herrmann, W. A. & Kocher, C. (1997). *Angew. Chem. Int. Ed. Engl.* **36**, 2162–2187.

[bb7] Melaiye, A., Simons, R. S., Milsted, A., Pingitore, F., Wesdemiotis, C., Tessier, C. A. & Youngs, W. J. (2004). *J. Med. Chem.* **47**, 973–977. 10.1021/jm030262m 14761198

[bb8] Sheldrick, G. M. (1996). *SADABS* University of Göttingen, Germany.

[bb9] Sheldrick, G. M. (2008). *Acta Cryst.* A**64**, 112–122. 10.1107/S0108767307043930 18156677

